# Me & My Wishes Videos: Congruence of End-of-Life Preferences Between Residents With Dementia, Family, and Staff

**DOI:** 10.1093/geroni/igaa057.2847

**Published:** 2020-12-16

**Authors:** Sarah Neller, Gail Towsley, Bob Wong

**Affiliations:** 1 University of Utah, Salt Lake City, Utah, United States; 2 University of Utah College of Nursing, Salt Lake City, Utah, United States

## Abstract

Me & My Wishes are person-centered videos of long term care residents (ages 65-95) living with dementia discussing their preferences for care including end-of-life (EOL) medical intervention. We evaluated the congruence of six EOL treatment preferences between the residents’ personal videos, medical records (e.g. advance directive), and surveys of family (n= 49) and staff (n=37; 118 responses) knowledge of their preferences. Results were highly discordant. Treatments with the most discordance when comparing videos to comparison groups were IV fluids (medical record, 57.1%) and life support (family, 69.4%; staff, 82.2%). Residents reported EOL treatments were considered acceptable if they were temporary, would relieve suffering, or enabled a return to baseline health. These caveats may lead to discordance if they are not conveyed to family or staff. Our findings highlight the need for conversations among residents living with dementia and their caregivers to improve understanding, congruence and adherence of resident EOL preferences.

